# Three-Dimensional Analysis of the *In Vivo* Motion of Implantable Cardioverter Defibrillator Leads

**DOI:** 10.1007/s13239-021-00557-4

**Published:** 2021-06-29

**Authors:** Tamas Szili-Torok, Jens Rump, Torsten Luther, Sing-Chien Yap

**Affiliations:** 1 Department of Cardiology, Erasmus MC, Postbus 2040, 3000 CA Rotterdam, The Netherlands; 2 Biotronik SE & Co. KG, Woermannkehre 1, 12359 Berlin, Germany

**Keywords:** Implantable cardioverter defibrillator, Right ventricular, Lead motion, Lead positioning, 3D reconstruction

## Abstract

**Abstract:**

Better understanding of the lead curvature, movement and their spatial distribution may be beneficial in developing lead testing methods, guiding implantations and improving life expectancy of implanted leads.

**Objective:**

The aim of this two-phase study was to develop and test a novel biplane cine-fluoroscopy-based method to evaluate input parameters for bending stress in leads based on their *in vivo* 3D motion using precisely determined spatial distributions of lead curvatures. Potential tensile, compressive or torque forces were not subjects of this study.

**Methods:**

A method to measure lead curvature and curvature evolution was initially tested in a phantom study. In the second phase using this model 51 patients with implanted ICD leads were included. A biplane cine-fluoroscopy recording of the intracardiac region of the lead was performed. The lead centerline and its motion were reconstructed in 3D and used to define lead curvature and curvature changes. The maximum absolute curvature *C*_max_ during a cardiac cycle, the maximum curvature amplitude *C*_amp_ and the maximum curvature *C*_max@amp_ at the location of *C*_amp_ were calculated. These parameters can be used to characterize fatigue stress in a lead under cyclical bending.

**Results:**

The medians of *C*_amp_ and *C*_max@amp_ were 0.18 cm^−1^ and 0.42 cm^−1^, respectively. The median location of *C*_max_ was in the atrium whereas the median location of *C*_amp_ occurred close to where the transit through the tricuspid valve can be assumed. Increased curvatures were found for higher slack grades.

**Conclusion:**

Our results suggest that reconstruction of 3D ICD lead motion is feasible using biplane cine-fluoroscopy. Lead curvatures can be computed with high accuracy and the results can be implemented to improve lead design and testing.

## Introduction

*In vivo* lead motion has a significant impact on lead performance. Therefore, it recently became a new focus in the regulatory field as well as in the field of implantable cardiac devices, as lead failure continues to be a crucial cause for malfunctions of the device system. The importance of lead motion analysis becomes especially evident when we look at recently reported lead-related complications such as lead externalization issues and fractures.^1^,^2^,^6^ Moreover, the development of a new engineering standard for cardiac rhythm management systems is in progress^5^ to formulate requirements on lead fatigue performance based on *in vivo* lead motion data. Currently, standards are based on established harmonized testing methods, but they do not take lead model specific use conditions, e.g. by *in vivo* lead curvatures and cyclic curvature changes, into account.

Routine static X-ray images are not able to assess such dynamic lead behavior and therefore cannot be used to analyze lead movements. Hoffman *et al*.^10^ showed that the determination of the three-dimensional *in vivo* positions of the leads is feasible by two synchronously acquired X-ray images with different view angles to minimize errors due to heart movements. A very limited number of studies are available assessing intracardiac *in vivo* curvature of leads.^4^,^8^,^14^,^18^

The aim of our study was to develop and test a novel method to analyze implanted defibrillator lead motion in three dimensions. The bending stress strongly depends on the curvature and the curvature amplitude, whereby the actual strain also depends on the structural properties of the lead and differ depending on lead type and manufacturer. The quantitative analysis of the specific stress was beyond the scope of this study.

## Methods

### Study Design

This study was a prospective, exploratory, non-randomized, single-center, feasibility study, and registered at the public national register of the Central Committee on Research Involving Human Subjects. Inclusion criteria were:

Patient has provided written informed consent; patient has a Biotronik ICD or CRT-D and at least a Biotronik Linox Smart S DX lead or any other Biotronik lead model; patient is able to attend the X-ray procedure following a routine follow-up visit; none of the leads was implanted within the last 3 months; patient had no cardiac intervention within the last 2 months.

Exclusion criteria were:

Patient age less than 18 years; patient is pregnant or breastfeeding; any complication of the implanted system at the time of enrollment.

The study was conducted in two phases. After developing a method to measure lead curvature and curvature evolution, it was initially tested in a phantom study. In the second phase using this method previously implanted leads were assessed. The Institutional MEC (Medical Ethics Committee) approved the study and all patients gave written informed consent. Patients with an implanted ICD- or cardiac resynchronization therapy defibrillator (CRT-D) system were enrolled during a regular patient follow-up at least 3 months after implantation of the device system. It was explicitly favored to enroll patients who received their ICD or CRT-D system from different implanting physicians. This was realized and the 51 patients were spread among 13 implanting electrophysiologists. A biplane cine-fluoroscopy procedure was conducted subsequent to the routine follow-up procedure. The cine-fluoroscopy window was limited to the intracardiac region, which ensured imaging of all electrically active elements of the leads (lead tip, shock coils, ring electrodes—including DX atrial dipole electrodes if applicable) during the full cycle length. The patients were in lying, i.e., horizontal position. No study-related follow-up procedure was required. Completion of the imaging and device follow-up was defined as the end of the study.

Although different biplane imaging systems are clinically approved and in use, a single-center design was chosen for this study to ensure efficient study execution and to avoid possible differences in imaging systems or system setups. The imaging was done with a Siemens Artis Zee biplane system together with the appropriate software.

In addition to the biplane cine-fluoroscopy images, data of the following categories were recorded: demographic data, medical conditions, type of implanted devices and leads, lead measurements at follow-up, X-ray procedural data, and adverse events during the study.

### Biomechanical Analysis

**Figure 1:**
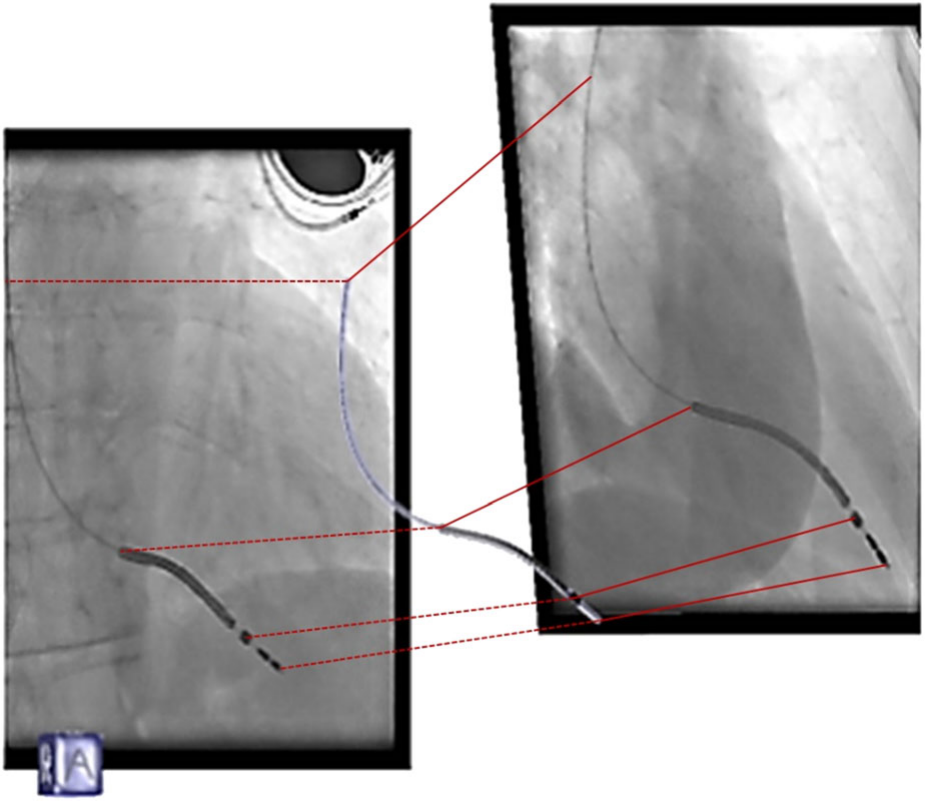
3D reconstruction of a lead from biplane cine-fluoroscopy. Left: LAO; and right RAO views with a minimum of 45 degrees difference.

The software tool, developed and successfully tested by Biotronik (Biotronik SE & Co. KG, Berlin, Germany), was used to reproduce the spatial (3D) lead movement from the biplane cinefluoroscopic imaging data (Fig. 1), and to precisely calculate curvatures and curvature changes of the lead. Version control was done via Lock Modify Write. The latest version was verified using artificial datasets based on *in vivo* data to determine the sensitivity and robustness. The assessment of the error, repeatability and reproducibility was done by the phantom study described further below.

**Figure 2:**
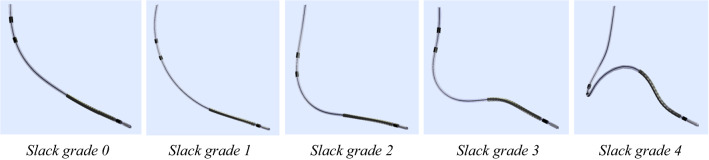
Slack classification. From left to right: grade 0: no slack, grade 1: minimal, grade 2: normal, grade 3: mildly excessive, and grade 4: very excessive slack.

The clinical data sets were used to assess the bending stress in leads based on the *in vivo* lead motion and deformation using associated modeling parameters, e.g. lead redundancy and curvatures. The lead redundancy was evaluated according to slack grades developed by the Ottawa Heart Institute.^6^ Slack grades for our classification are exemplified in Fig. 2.

Curvatures were calculated from 3D reconstruction of the lead centerline. The reconstruction was performed for all recorded frames of a cardiac cycle (30 frames/s). The intracardiac reconstruction length was at least 15 cm starting from the distal end to capture the locations of the maximum intracardiac curvature and curvature amplitude.

The intracardiac lead curvature was analyzed by the following parameters (see nomenclature):

$$C_{\hbox{max} } ,\;C_{\text{amp}} ,\;C_{\hbox{max} } @C_{\text{amp}} ,\;s\left( {C_{\hbox{max} } } \right),\;s\left( {C_{\text{amp}} } \right)$$

**Figure 3:**
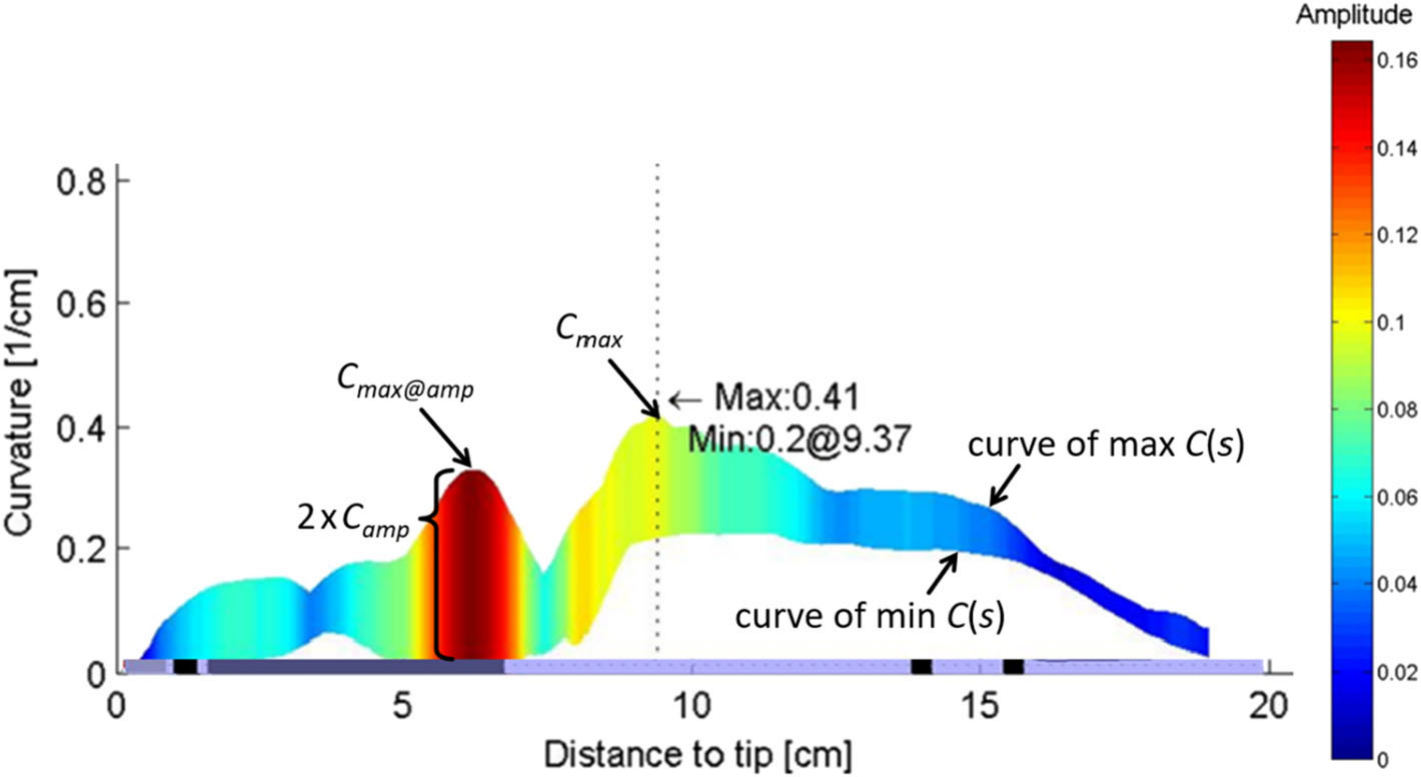
Illustration of estimated curvatures and curvature amplitudes calculated from 3D ICD lead centerline reconstruction.

Figure 3 illustrates the most important parameters of the curvature analysis. First, at each discrete time step (*t*) of the cardiac cycle and at each discrete distance to tip (*s*) the lead centerline curvature was calculated. From that, curves of the maximum curvature during the cardiac cycle (max *C*(*s*)) and the minimum curvature during the cardiac cycle (min *C*(*s*)) could be provided for the reconstructed intracardiac region. The maximum of max *C*(*s*) for all *s* yielded the maximum absolute curvature *C*_max_ that occurred during a cardiac cycle in the reconstructed lead. *C*_max_ is a measure for the highest static bending stress in the lead. The maximum curvature amplitude *C*_amp_ is a measure for the highest dynamic bending stress in the lead. Additionally, the maximum curvature *C*_max_*@C*_amp_ at the location of *C*_amp_ was determined from max *C*(*s*). Both *C*_amp_ and *C*_max_*@C*_amp_ can be used to characterize the relevant intracardiac fatigue stress in a lead under cyclical bending. Leaving potential unknown external forces aside, static curvature of the lead has a smaller influence on the lead’s lifetime than dynamic changes within the observed range of curvatures. Therefore, a combination of the maximum absolute curvature at any position with the maximum curvature amplitude may overestimate the mechanical *in vivo* bending stress of the lead. Therefore, pairing the maximum curvature amplitude and the maximum curvature at the location of the maximum curvature amplitude could be a better measure for the dynamic bending stress a lead is exposed to.

Finally, the locations of *C*_max_ and *C*_amp_ on the reconstructed lead were analyzed by their distance to tip (*s*(*C*_max_) and *s*(*C*_amp_)). Often, *C*_max_*@C*_amp_ equals *C*_max_. This means that the maximum absolute curvature occurs at the location of *C*_amp_.

Prior to the patient analysis, the uncertainty of the curvature estimation of the software tool developed by Biotronik was analyzed regarding its reproducibility and repeatability via a phantom study.

### Phantom Study: Uncertainty Analysis

Two 3D lead paths (phantom 1 and phantom 2) based on *in vivo* lead paths were constructed with four known local maximum curvatures each. The curvatures were given by planar path segments composed of a fourth-order polynomial of the form

$$y = ax^{4} + bx^{3} + dx$$

**Figure 4:**
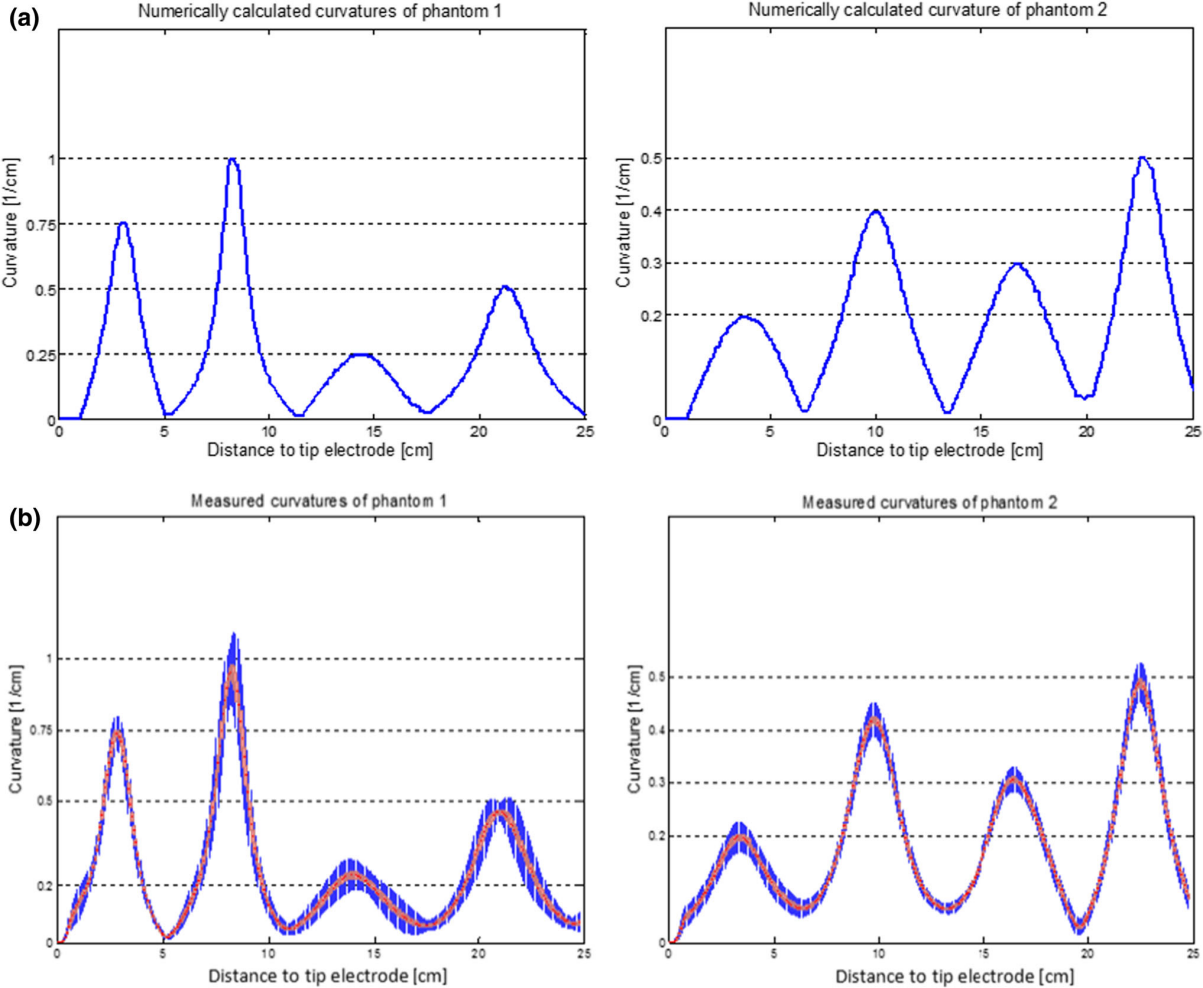
Results of the phantom study. (a) Spatial distribution of the curvature of phantom 1 (left) and phantom 2 (right). The values were calculated numerically for the theoretical lead path. (b) Whiskers plot of all reconstructed curvatures of phantom 1 (left) and phantom 2 (right). The red marks represent the median of the spatial distribution of the curvature.

The local maxima of the curvature of phantom 1 were: 0.75, 1.0, 0.25, and 0.5 cm^−1^. Phantom 2 had the local maximal curvatures: 0.2, 0.4, 0.3, and 0.5 cm^−1^ (Fig. 4). The planar segments were rotated in space and combined to form a three-dimensional path with curvatures of zero at the junctions. The coordinates of these synthetic lead paths were used to build a phantom body with a 3D printer, with channels in the body to place Linox Smart SD 65/16 leads, manufactured by Biotronik, in a stable position. The bodies of phantom 1 and phantom 2 were designed by Biotronik and printed with acrylic glass powder by an external supplier.

Biplanar X-ray images of phantom 1 and phantom 2 were acquired at two different angulations and different positioning of the phantoms. The imaging system was the same Siemens Artis Zee Biplane at the Erasmus Medical Center Rotterdam that was used for image acquisition from patients in the second (patient) phase of the study.

The user interface for the determination of the imaged lead path was programmed with Matlab R2013b. The optimization to determine the geometry of the imaging system was based on the Nelder-Mead algorithm.^13^ After identifying associated image pixels of the lead path in the two views, the 3D coordinates of the path were calculated according to epipolar geometry.^9^ The 3D coordinates were smoothed by a cubic spline based on the Fortran function Smooth.^15^ The curvature was piecewise calculated with a sliding window of 5 mm width.

To assess the reproducibility of the localization of the lead path in the images, 5 different operators (referred as “identifier”) manually marked the position of the lead paths. This localization was done with 5 images of each phantom (2) and view (2) to determine the repeatability. Thereby, a total of 100 pairs of biplane images were evaluated. The optimization and reconstruction were done by 2 different operators (referred as “reconstructor”) to determine the reproducibility of the post processing.

### Patient Phase: *In Vivo* Three-Dimensional Lead Imaging and Reconstruction

The imaging procedure was scheduled in conjunction with a routine patient and device follow-up visit. During the procedure, the cine-fluoroscopy field of view was limited to the intracardiac region to focus on all electrically active elements of the leads, i.e. lead tip, shock coils, and ring electrodes including DX atrial dipole electrodes of Biotronik Linox smart S DX leads. All images had to fulfill the following requirements:

Frame rate of 30 frames/s; the recording duration is about 4 s to acquire at least 3 entire cardiac cycles; the angulation between the two biplane views must be greater than 30°, at best 90°; and the complete intracardiac lead region is visible in both biplane images during the entire cardiac cycles.

The cine-fluoroscopy was performed by a trained team led by the corresponding author. The following steps were elements of the examination procedure for a particular patient after enrollment: admission to the lab equipped with the biplane fluoroscopy, patient on the table and placing standard ECG leads. Then positioning of the imaging system to ensure appropriate biplane views and verification of frame rate. It was followed by cine-fluoroscopy while the patient holds his/her breath. Breath hold was preferred to ensure that the entire lead portion remained within the field of view during imaging. Before the patient left the room, we verified the acquired images and proper storage of the data. If a second cine-fluoroscopy was necessary, we rearranged the imaging system and/or patient position. Thereafter, ECG and the supportive systems were removed. At the end of the procedure, the patient was released and the data were archived as DICOM RAW data.

### Statistical Analysis

Exploratory data analyses e.g. age, gender and heart condition, were used to describe the patient population. The sample size was defined to be at least 45 eligible imaging data sets from enrolled patients. This sample size was defined based on an estimation to acquire a feasible number of imaging data sets within a normal range of lead motion patterns and heart anatomies. Finally, clinical data sets have been acquired from 51 patients, and intracardiac lead curvatures and assessment of lead slack were statistically analyzed. Pearson’s correlation coefficient was used to measure the correlation between slack grade and maximal lead curvature amplitude and maximum curvature at the location of maximum curvature amplitude. During the phantom analysis the following statistical methods were applied: since the mechanical stress is mainly due to high curvatures with high changes of amplitude over the heart cycle, the main focus of the analysis was on the local maxima of the curvature. A statistical analysis, including an analysis of variance (ANOVA), was performed.

## Results

### Patient Population

The patient population consisted of 51 patients in routine medical care with an implanted Biotronik ICD or CRT-D device and Biotronik leads. None of the patients had any cardiac intervention within the last two months prior to the study. All patients had ICD leads with active fixation in the right ventricular apical region. The mean age of the patients was 56.4 ± 13.6 years (ranging from 18 to 83 years). Thirty-four (67%) male and 17 (33%) female patients were included. Presence of heart failure was reported in 19 (37%) patients, 6/19 (32%) were in NYHA class I, 12/19 (63%) in NYHA class II, and 1/19 (5%) in NYHA class III. Recent measurements on left ventricular ejection fraction were available for 31 patients, showing a mean value of 39% (ranging from 20% to 71%).

### Phantom Study

The local maxima of the reconstructed curvature were in good agreement with the theoretical curvature distribution (Fig. 4). The mean error (bias) over all curvature maxima was 3.6 ± 11.3%. The estimated curvature showed a tendency to overestimate the theoretical curvature, especially for the curvature 1.0 cm^−1^ (bias 4%). The maximal relative variance occurred at a curvature of 0.25 cm^−1^ with 18.5%. The most relevant results of the ANOVA are summarized in Table 1. The main influence on the variance was the repetition of the curvature estimation (> 90%). The different identifiers, reconstructors and amplitudes of the local curvature maxima had only a minor effect on the variance.

**Table 1:** Results of the ANOVA on phantoms 1 and 2 with the reconstructor (Operator) and local maxima of curvature.

Source	Standard deviation	% of total variation
Repeatability	0.109	92.03
Operator	0.000	0.00
Interaction (OP)	0.018	2.36
Reproducibility	0.018	2.36
Total gage R&R	0.111	94.39
Part-to-part	0.027	5.61
Total variation	0.114	100.00

*Part* also referred as “arch” as parameter of variance

### Patient Study

**Figure 5:**
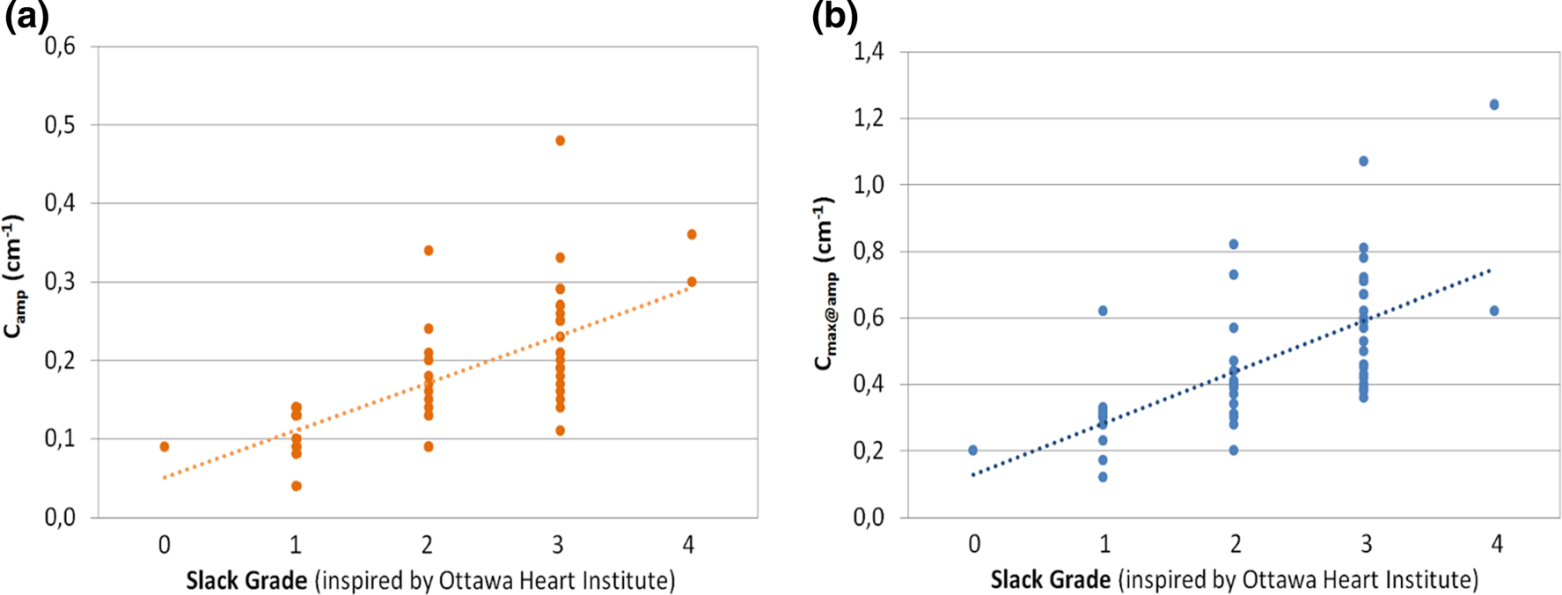
(a) correlation between slack grade and maximum curvature amplitude. (b) correlation between slack grade and maximum curvature at location of maximum curvature amplitude.

The slack grade distribution was analyzed for all ICD leads included in our study. All leads had an apical tip location. The mean slack grade was 2.3 ± 0.9. While all slack grades were present in our study, grades 3 (45%) and 2 (29%) were most frequently observed. Maximum lead curvature amplitudes (correlation *r* = 0.64, *p* value = 4.e-7) and maximum curvatures at the location of maximum curvature amplitude (correlation *r* = 0.63, *p* value = 8.e-7) were found to significantly increase with lead slack. These correlations are illustrated in Fig. 5.

**Figure 6:**
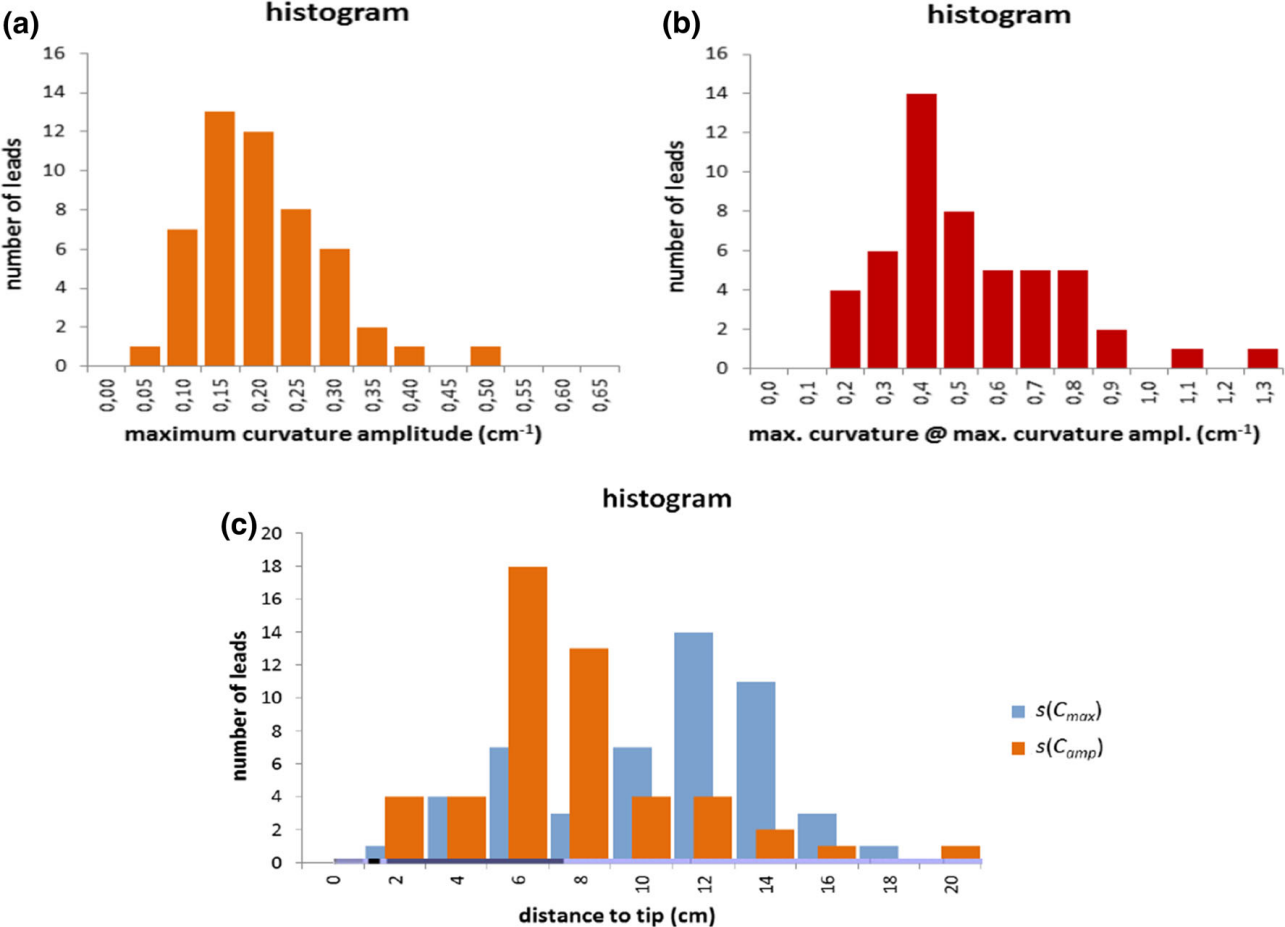
(a) frequency distribution of the maximum curvature amplitude. (b) frequency distribution of the maximum curvature at the location of the maximum curvature amplitude. (c) frequency distribution of the location of maximum curvature *s*(*C*_max_) and maximum curvature amplitude *s*(*C*_amp_).

Considering all 51 right ventricular ICD leads the median of the maximum curvature amplitude *C*_amp_ was 0.18 cm^−1^. The frequency distribution of this parameter can be seen in Fig. 6a. The median *C*_amp_ was 0.18 cm^−1^ (Q1–Q3 0.13–0.25, range 0.04–0.48). The median C_max@amp_ was 0.42 cm^−1^ (Q1–Q3 0.33–0.62, range 0.12–1.24). The curvature 0.42 cm^−1^ was also the curvature with the highest count in the patient data (Fig. 6b). As expected, this median value was considerably lower than the median of the overall maximum curvature of 0.53 cm^−1^.

The observed locations of maximum absolute curvature *C*_max_ and maximum curvature amplitude *C*_amp_ were spread over the whole intracardiac region and were equal in about 45% of analyzed ICD leads. The median location of *C*_max_ was in the proximal region of the intracardiac lead segment (median 10.7 cm, Q1–Q3 6.6–12.3, range 1.7–17.9), which is most likely in the atrium, whereas the median location of *C*_amp_ occurred at a more central region of the intracardiac lead segment (median 6.0 cm, Q1–Q3 4.8–8.0, range 1.3–18.5) close to where the transit through the tricuspid valve can be assumed. The frequency distributions of *s*(*C*_max_) and *s*(*C*_amp_) are shown in Fig. 6c.

### Device Interrogation After the Patient Study

**Figure 7:**
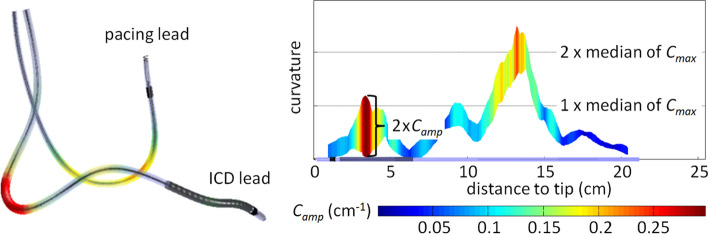
ICD lead with very excessive slack and malfunction. Left: 3D reconstruction of leads with highlighted maximum curvatures. Right: envelope of curvatures along the ICD lead.

During ICD interrogation two lead-related problems were detected, one at 2 months and the other at 26 months after the end of the study. One patient experienced lead dislodgement requiring repositioning. The ICD lead had minimal slack (Ottawa slack grade 0) and a relatively low *C*_max_ = 0.6 × median of *C*_max_ (median of all 51 patients). The other patient had lead dysfunction (oversensing). The ICD lead had excessive intracardiac slack and a high *C*_max_, 2.4 x median of *C*_max_ (Fig. 7). The location of highest *C*_max_ was in the right atrium, which also demonstrated a relatively high *C*_amp_. The patient later underwent a full system extraction.

## Discussion

We developed a novel method to analyze implanted defibrillator lead motion in three dimensions. The major finding of this initial evaluation is that this assessment based on biplane fluoroscopy recording is feasible, highly accurate and can be implemented for the assessment of the intracardiac *in vivo* movement of right ventricular implantable cardioverter-defibrillator (ICD) leads.

### Lessons Learned from the Phantom Study

Several studies regarding the calculation of a digital path’s curvature in computer vision in 2D have shown that it is very challenging to keep the curvature error below 10% and errors of 30–50% are not unusual.^11^,^16^,^17^ The challenges increase even more in 3D space.^7^ In order to overcome these challenges in this study, the 3D coordinates were calculated using epipolar geometry in combination with an applied smooth cubic spline. As a result, the curvature was determined by solving a linear equation with multiple coordinates in two dimensions. Obviously, due to these multiple steps in the post processing, our analytical approach to estimate the curvature error became very complex. Because of the limited contrast of X-ray images compared to optical images the identification of the lead path was performed manually. This manual selection of the lead path is more adaptive to changes in contrast and image noise although it may be prone to spatial errors of the reconstruction calculation. On the other hand, during reconstruction of the 3D points of the lead path, the use of a parametric function describing the run of the path is a crucial factor for the curvature estimation. Therefore, the underlying function used in the software was continuously differentiable up to the second derivation to avoid breaks at the junctions of the function segments. This allowed avoiding smoothing in the post processing such as spatial averaging.^14^ Our data clearly demonstrate that the variance is mainly due to the variance of repeatability. The individual identifier/reconstructor or parameters like image geometry and amplitude of curvature had only a negligible influence. Of note, large out-of-plane lead segments increased the impact of errors made during the manual localization of the imaged lead path. Therefore, the determination of the reconstruction error was assumed to be a worst-case error scenario. Taking this into account, the uncertainty of our method relating the curvature estimation was comparatively low (3.6% mean overestimation, 11.3% SD) and hence suitable for the determination of *in vivo* curvatures.

### Patient Study Reveals Potential Lead Positioning Issues

The values published by Baxter *et al*.^3^ with maximum intracardiac curvatures from 0.27 to 0.78 cm^−1^ and mean maximum curvature 0.48 cm^−1^ (0.18 cm^−1^ SD) for 20 patients are comparable to the data shown in our study. A curvature of 1.4 cm^−1^ reported by Liu *et al*.^14^ was based on a single patient with a dataset taken from a prior study,^18^ with a position of the tip of the pacing lead in the right ventricular outflow tract. The clinical data demonstrate that increased intracardiac slack was associated with higher static and dynamic lead stress as expressed as maximum curvature at the location of maximum curvature amplitude, and maximum curvature amplitude. This may imply that excessive intracardiac slack may negatively impact lead survival. This is highlighted by the patient in our study who required lead extraction after the detection of noise (Fig. 7). Interestingly, a previous case–control study demonstrated that excessive intracardiac slack was associated with lead conductor fracture in Medtronic Fidelis 6949 leads.^12^ Previously, the dynamic nature of slack was poorly investigated. The constructed model provides insight into the location of potential mechanical lead stress, which is in the atrium for maximum curvature and at the level of the tricuspid valve for the maximum curvature amplitude. This information can be used to facilitate the development of more durable leads.

### Clinical Implications and Limitations

Using our methodology may open future perspectives. Previous trials failed to accurately reconstruct three-dimensional lead behavior *in vivo*. The data derived from our phantom study and the clinical evaluation are very promising and may pave the road for future possible clinical implementation. Although we have no follow up data, it is very appealing that the patient with the most excessive loop and lead stress had lead dysfunction. It raises the possibility of implementing this as a guiding tool for implantation. Obviously, the major limitation is the limited availability of biplane fluoroscopy in the EP lab. However, the data can be used to initiate adjustments in the regulatory process and more adequate testing methods can be developed. Moreover, during lead design the susceptible regions can be identified and strengthened and may result in the development of more durable leads.

## Conclusions

In conclusion reconstruction of 3D lead motion is feasible using biplane cine-fluoroscopy and lead curvatures can be computed with high accuracy. An increase of dynamic stress with increasing slack was confirmed. Our results can be implemented to improve lead design and testing. In the future even lead positioning can be assisted using slack assessment as a guiding tool and it may provide useful information before lead extraction procedures.

## Abbreviations

N: Number
SD: Standard deviation
Min: Minimum
Max: Maximum
Q1: First quartile
Q3: Third quartile
*S*: Distance to lead tip (along the lead); the lead tip equals the tip of the extended helix
max *C*(*s*): Maximum curvature of the lead centerline during cardiac cycle at a particular distance to tip
min *C*(s): Minimum curvature of the lead centerline during cardiac cycle at a particular distance to tip
*C*_max_: Maximum absolute curvature of the lead centerline during cardiac cycle
*C*_amp_(*s*): Curvature amplitude during cardiac cycle at a particular distance to tip; *C*_amp_(*s*) = [max *C*(*s*) − min *C*(*s*)]/2
*C*_amp_: Maximum curvature amplitude of the lead centerline
*s*(*C*_max_): Distance of *C*_max_ location to lead tip
*s*(*C*_amp_): Distance of *C*_amp_ location to lead tip
